# Validation of a convolutional neural network that reliably identifies electromyographic compound motor action potentials following train-of-four stimulation. Comment on *Br J Anaesth Open* 2023; 8: 100236

**DOI:** 10.1016/j.bjao.2024.100264

**Published:** 2024-02-22

**Authors:** Willis Silliman, Zain Wedemeyer, Srdjan Jelacic, Andrew Bowdle, Kelly E. Michaelsen

**Affiliations:** Department of Anesthesiology and Pain Medicine, University of Washington, Seattle, WA, USA

**Keywords:** compound motor action potential, electromyography, machine learning, neural networks, noise-filtering algorithm

Editor—A recent article by Epstein and colleagues^1^ described the use of a neural network for the identification of compound motor action potentials during electromyographic-based quantitative neuromuscular block monitoring. We agree with the authors that managing electrical noise^2^ is essential for effective electromyography-based monitoring. The ability of the twitch monitor to discern whether or not the output generated by the device after electrical stimulation corresponds to a twitch is essential to ensure accurate train-of-four and post-tetanic counts. Whether using neural networks provides additional benefit to determining train-of-four and post-tetanic counts when compared with palpation or noise management algorithms used in commercially available electromyography-based twitch monitors is unclear. There are three issues which we would like to highlight that could limit the impact of this work: (1) lack of specific characterisation of small amplitude signals, (2) lack of comparison to a validated assessment strategy, and (3) issues with the machine learning methodology.

Regarding the first point, the authors tested the algorithm on signals from patients undergoing elective operations with spontaneous recovery from neuromuscular block, in which they note there were much higher signal amplitudes than in the training group undergoing laparoscopic or robotic surgery. They acknowledge that although ‘the algorithm works extremely well when the signal amplitude is large’, it would be more relevant to report the performance of the network when signal amplitudes are small. Rather than reporting aggregate results including a large number of high train-of-four ratios, a breakdown by signal amplitude could be more convincing. Additionally, mixing or swapping the original testing and training sets would increase the number of low amplitude signals in the testing set for assessment.

Secondly, the authors chose visual inspection of the electromyography waveforms to determine twitch validity, an approach which has never been validated. Comparison to gold standard mechanomyography or more simply, palpation would have been much more convincing. Palpation is a traditional method that is familiar to clinicians. We and others have previously demonstrated that palpation has a substantial agreement with electromyography and mechanomyography when assessing train-of-four counts.^3^^,^^4^

Lastly, in its current state, the proposed neural network would be difficult to run on the processor in an electromyograph without significantly increasing the monitor cost. Recommendations on how to make a neural network more suitable include use of a convolutional neural network, one-dimensional data representation, balancing the training class, and k-fold tuning as detailed below.

It is unclear what network design was used in this study as the code was not made available. The authors describe a convolutional neural network, but the structure presented in Figure 3 does not resemble one.^5^ Figure 3 represents a basic, fully connected neural network which does not use convolutional layers and instead weighs every activation of the previous layer into every node in the next layer. Figure 1 shown here illustrates the difference between the two network types. A fully connected network is less suitable for this application as it takes longer to learn the relationship between pixel clusters, because this relationship is not inherently built into the network. It is also less efficient because of the higher dimensionality of the model which explains why the authors ran into computation issues while fitting the model.

**Fig 1:**
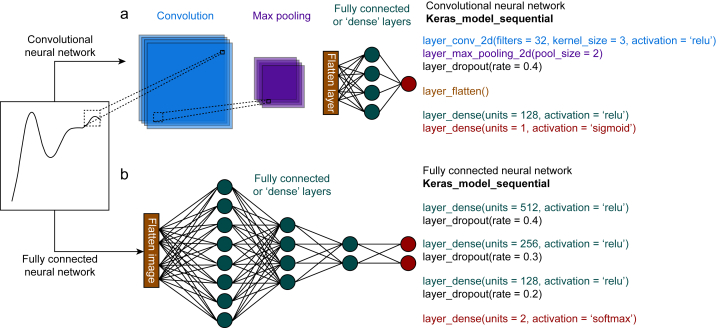
A visualisation of the difference between convolutional and fully connected neural networks. (a) An example of convolutional neural networks which passes a series of filters over an image. (b) An example of fully connected neural network. On the right of each panel image, we give an example of how to generate a convolutional network using the Keras library in R, which is different from the code given by the authors in Figured 3 of their paper. Because of convolution and max pooling steps, the number of nodes is greatly reduced at the flatten layer step shown for the convolutional neural network when compared with the fully connected neural network, leading to greater efficiency, and reduced computational requirements.

By using a one-dimensional data representation, the authors would have avoided the need to discard data points beyond certain thresholds and the need to smooth the waveforms. The compound motor action potential data consisting of measurements equally spaced in time can be easily represented in one dimension. There are several other choices of neural network models including one-dimensional convolutional models^6^ and recurrent neural networks^7^ that can perform similar electric signal classification tasks efficiently. Network complexity was also unnecessarily increased by using two output nodes for a binary classification task which can be handled by a single node, as only two output categories are used (valid response and noise).

The training dataset is imbalanced as less than a quarter of the data points were valid responses resulting in a biased model that would tend to incorrectly predict valid responses as noise. When testing the model's performance on easily recognisable twitches, the accuracy would be high (as reported). However, the imbalanced data would likely lower the model's accuracy if more ambiguous twitches were reported (such as post-tetanic counts).^8^

The authors state that leave-one-out cross-validation was used to tune model hyperparameters for training the dataset, which implies that a single data point was removed from the dataset to fit a model. This would then be repeated for each data point. However, it appears that the authors instead used leave-one-subject-out cross-validation where all compound motor action potentials from one subject are left out rather than a single data point. Although this validation method may account for bias associated with specific patients, others have demonstrated that leave-one-subject-out cross-validation tends to inflate reported accuracy when compared to k-fold cross-validation.^9^ It appears that k-fold was only used to validate the model and not to tune it despite k-fold being a better model selection technique. Additionally, the authors state that image size was decreased to make hyperparameter tuning and cross-validation faster. This changes the network design as the input dimension is significantly smaller, meaning there are fewer weights between the input and first hidden layers. The hyperparameters that worked best for this network may be suboptimal for the larger network. Furthermore, this invalidates the accuracy metrics reported in Table 2 as useful measures of the proposed model. The input dimension of a fully connected model cannot be reduced with the expectation that the resulting networks will behave in the same way.

The application of neural networks to improve assessment of train-of-four and post-tetanic counts is an innovative approach worthy of consideration. However, integrating neural networks into electromyography-based monitors is not trivial as it requires additional computing power and hardware. Most importantly, the benefit of adding neural networks to current noise management algorithms should be demonstrated by comparing such monitoring systems with palpation and existing noise management algorithms, especially at deeper levels of neuromuscular block when signals are small.

## Declarations of interest

All authors declare that they have no conflicts of interest.

## Funding

KEM's research is supported by the 10.13039/100005831Foundation for Anesthesia Education and Research and the 10.13039/100001906Washington Research Foundation. AB's research is supported in part by the Laura Cheney Professorship in Anesthesia Patient Safety.
